# Relationships between Multimorbidity and Suicidal Thoughts and Plans among Korean Adults

**DOI:** 10.3390/jcm8081094

**Published:** 2019-07-24

**Authors:** Youn Huh, Ga Eun Nam, Yang-Hyun Kim, Jun Hyung Lee

**Affiliations:** 1 Department of Family Medicine, Inje University Ilsan Paik Hospital, College of Medicine, Inje University, 170, Juhwa-ro, Ilsanseo-gu, Goyang-si, Gyeonggi-do, Seoul 10380, Korea; 2 Department of Family Medicine, Korea University Anam Hospital, Korea University College of Medicine, 73, Goryeodae-ro, Seongbuk-gu, Seoul 02841, Korea

**Keywords:** chronic disease, multimorbidity, suicidal thoughts, suicidal plans

## Abstract

Multimorbidity and suicide rates are rising simultaneously among Korean adults. To address this issue, we assessed the association between multimorbidity and suicidal behavior among adults aged ≥19 years in Korea. We analyzed the data from the 2017 Korea National Health and Nutrition Examination Survey. Multimorbidity was defined as experiencing two or more chronic diseases. We compared the presence of suicidal thoughts and plans according to multimorbidity using chi-square test, and examined the associations between multimorbidity and suicidal thoughts and plans using multiple binary logistic regression analyses. Multimorbidity was found in 30.8% of total participants. As the number of chronic diseases increased, the percentage of thoughts and plans tended to increase (*p* < 0.001 and *p* = 0.002). Among participants with multimorbidities, 8.5% had suicidal thoughts, whereas only 3.4% without multimorbidity had such thoughts (*p* < 0.001). Participants with multimorbidity had significantly higher odds of suicidal thoughts (OR = 2.14; 95% CI = 1.54–2.97) and suicidal plans (OR = 2.01; 95% CI = 1.08–3.73) compared to those without multimorbidity after adjusting confounding variables. **Conclusion:** People with multimorbidity had a higher prevalence of suicidal thoughts and plans. Early detection of and intervention for suicidal thoughts and plans are critical for suicide prevention among people with multimorbidity.

## 1. Introduction

The National Commission on Chronic Illness in the United States defines chronic illness as permanent illnesses accompanied by disability due to complication or injury, and those requiring special training for rehabilitation, long-term protection, monitoring, and treatment [1]. These individuals require continuous and comprehensive medical intervention, and communication and self-care is necessary [2]. Multimorbidity refers to the state of having multiple chronic illnesses simultaneously, potentially mixed with acute illnesses as well [3]. Unlike the concept of co-morbidity, which refers to how the impact of indicator diseases are influenced by co-morbid illnesses, the concept of multimorbidity is firmly centered on individuals with multimorbidity [4].

There has been an increase of chronic illnesses following the extension of longevity in society. As expected, in South Korea the prevalence of chronic illnesses and multimorbidity has been shown to increase with age. An analysis of older adults with one or more chronic illnesses revealed that 70.9% of older adults with chronic illnesses have multimorbidity, reaching an average of 4.1 chronic illnesses [5]. Another study found that older patients with multimorbidity had an average of 5.1 chronic illnesses, whereas patients without multimorbidity tended to have an average of 1.6 [5]. There have been several studies on the definition of multimorbidity [3,6]. Because people with two or more chronic medical conditions are more common than those with three or more chronic medical conditions, former status is more frequently accepted as the definition of multimorbidity [6]. Studies adopting this definition reported that multimorbidity better reflected poor quality of life, impaired functioning, and increased use of medical facilities such as emergency admissions, particularly with higher numbers of coexisting conditions [7,8,9,10,11].

Suicide is one of the leading causes of death worldwide [12]. Among the countries in the Organization for Economic Co-operation and Development (OECD) as of 2012, the suicide rate (per the OECD standard population of 100,000 people) in South Korea was 29.1, which is the highest out of all OECD countries (average is 12.5). The specific risk factors associated with suicide among adults include depression and other mental disorders, illnesses, loss of function, the death of a partner, and other traumatic incidents [13]. By comparison, according to a 2006 report by the World Health Organization (WHO), the general risk factors of suicide include low socioeconomic status, education level, loss of a job, social stress, mental disorders, illnesses, and chronic pain [14,15]. One study found that 34% of people with suicidal thoughts have plans for suicide, 72% of people who plan for suicide actually attempt suicide, and 26% of people who have suicidal thoughts attempt suicide impulsively without having elaborated a prior plan to do so [16]. Therefore, suicidal thoughts and suicidal plans are important when discussing suicide.

Multimorbidity is associated with suicide-related risk, and primary care and mental health clinics need to evaluate for suicide ideation for patients with multimorbidity [17]. To address the impact of these issues, we aimed to investigate the increase in suicide-related variables in multimorbidity among Korean adults. Specifically, we examined the prevalence of suicidal thoughts and plans among adults who participated in the Korea National Health and Nutrition Examination Survey (KNHANES) in 2017, depending on whether they had multimorbidity.

## 2. Methods

### 2.1. Participants and Data

Drawing on raw data from the second year of the 7th KNHANES of 2017, we initially selected a total of 8127 individuals. Among them, we excluded people aged ≤18 years (*n* = 1938) and those who had any missing variables (*n* = 395), because people under the age of 18 did not perform suicide-related questionnaire in the 7th KNHANES of 2017. Finally, 5794 adults were considered in our analyses.

The KNHANES is a cross-sectional health surveillance system that assesses the status of and trends in national health and nutrition of South Korea to identify vulnerable groups to be prioritized in health policies, as well as evaluate whether existing health policies and projects are effective. It provides data on smoking habits, drinking habits, physical activities, and obesity, as well as various other statistics required by the WHO and OECD. The KNHANES comprises a health interview survey, a nutrition survey, a health examination survey, and data on demographic characteristics, diet, and health collected through personal interviews. Physical examinations along with blood and urine sampling were carried out at a mobile examination center. A stratified, multistage probability sampling design was used to select the household units that participated in the survey [18].

Our study adhered to the principles of the Declaration of Helsinki and was approved by the Institutional Review Board of Ilsan Paik Hospital (No. ISPAIK IRB 12 February 2017). Requirement of informed consent was waived because anonymous and de-identified information was used.

### 2.2. Research Variables

The chronic illnesses surveyed in the 7th KNHANES included hypertension, dyslipidemia, cerebral infarction (stroke), myocardial infarction, angina, osteoarthritis, rheumatoid arthritis, osteoporosis, tuberculosis, asthma, allergic rhinitis, depression, renal failure, atopic dermatitis, diabetes mellitus, thyroid disease, stomach cancer, liver cancer, colon cancer, breast cancer, cervical cancer, lung cancer, thyroid cancer, other cancers, hepatitis B, hepatitis C, and cirrhosis. Participants who self-reported not currently having a disease or a doctor’s diagnosis of a disease were regarded as not having it; by contrast, participants who self-reported having a disease or a doctor’s diagnosis of one were defined as having a chronic illness. Of these, stomach cancer, liver cancer, colon cancer, breast cancer, cervical cancer, lung cancer, thyroid cancer, and other cancers were combined into an overarching “cancer” category.

In the mental health section of surveys, participants were asked to answer the following questions (employing answer options of yes, no, or I do not know/no answer): “Have you seriously considered suicide in the last year?” and “Have you seriously planed suicide in the last year?”

People who had smoked at least 100 cigarettes in their lifetime and continued smoking at the time of survey were defined as current smokers. People who consumed at least one alcoholic beverage per month in a year prior to the survey were considered alcohol drinkers and those who did not were considered non-drinkers. Education level was categorized into two groups depending on whether participants had achieved a higher qualification than middle school graduation or not. Monthly personal income level was divided into two groups: the lowest quartile group and the second-lowest to highest quartile group.

### 2.3. Analysis Method

We combined the raw data from the 2017 KNHANES according to the KNHANES raw data analysis guidelines. Based also on a complex sample design, we conducted all analyses by assigning a dispersed stratification estimation, stratification variables, and weighted sample values. We divided participants who had 2 or more of the above-stated chronic illnesses as multimorbidity based on the definition used in the previous study [6]. Subsequently, a chi-square test was performed to evaluate proportions of suicidal thoughts and plan according to the number of chronic illness or the presence of multimorbidity. Then, to determine the association of suicidal thoughts and plans with multimorbidity, we conducted a multiple binary logistic regression analysis and calculated the odd ratio (OR) and 95% confidence intervals (CIs). Model 1 did not adjust. Model 2 adjusted for sex and age. Model 3 adjusted for sex, age, education, personal income, smoking status, and alcohol consumption. Since there are studies showing that income and education level are related the risk of suicide, we adjusted these variables [14,15]. Stratified analyses according to sex and age were also performed. All analyses were performed using SPSS Statistics 21.0 (IBM Corp., Armonk, NY, USA).

## 3. Results

### 3.1. Participants’ General Characteristics

Participants’ demographics are shown in Table 1. A total of 5794 adults were included. Of the entire sample, 30.8% had multimorbidity. The proportion of patients with multimorbidity increased with age. The proportion of women with multimorbidity was higher than that of men. In addition, the ratio of people with multimorbidity was higher when education level or income was low. Regarding smoking, the percentage of patients with multimorbidity was the highest among ex-smokers. The proportion of patients with multimorbidity was higher in non-drinkers than in drinkers.

### 3.2. Suicidal Thoughts and Plans According to the Number of Chronic Diseases

Regarding suicidal ideation, 5.0% of participants had suicidal thoughts and 1.3% had suicidal plans. In the absence of chronic diseases, suicidal thoughts were present in 2.7% of the cases, and suicidal plans were present in 0.6%. If the patients had one chronic disease, suicidal thoughts were present in 4.5% of the cases, and suicidal plans were present in 1.6%. Suicidal thoughts and plans were present in 9.7%, and 2.2% of participants who had more than 3 chronic diseases, respectively. As the number of chronic diseases increased, the proportions of suicide thoughts and plans tended to increase (*p* < 0.001 and *p* = 0.002) (Table 2).

### 3.3. Suicidal Thoughts and Plans According to the Presence of Multimorbidity

Table 3 shows the relationship between multimorbidity and suicidal thoughts and plans. While 3.4% of adults without multimorbidity were found to have suicidal thoughts, 8.5% of adults with multimorbidity had such thoughts (*p* < 0.001). As for suicide plans, 2.1% of adults with multimorbidity and 1.0% without multimorbidity were found to have them, respectively (*p* = 0.003).

### 3.4. Multivariable Logistic Regression Analysis Between Multimorbidity and Suicidal Thoughts and Plans

Table 4 presents multivariable logistic regression analysis results regarding the association of multimorbidity with suicidal thoughts and plans. Compared to participants without multimorbidity, participants with multimorbidity had significantly higher odds of suicidal thoughts in all adjusted models (OR, 95% CI: 2.65, 1.95–3.60 in model 1; 2.49, 1.82–3.40 in model 2; 2.14, 1.54–2.97 in model 3). Stratified analysis with sex and age showed similar findings. In men, the odds of suicide thoughts increased by 3.1 times among people with multimorbidity compared to those without multimorbidity after adjusting for all potential variables (OR, 95% CI: 3.09, 1.84–5.20 in model 3). The odds of suicide thoughts increased by 3.2 times in younger people (19–40 years) with multimorbidity compared with those without it (OR, 95% CI: 3.15, 1.43–6.97 in model 3). For suicidal plans, compared to participants without multimorbidity, participants with it had higher chances of suicidal plans (OR, 95% CI: 2.14, 1.30–3.54 in model 1; 2.26, 1.22–4.20 in model 2; 2.01, 1.08–3.73 in model 3). Suicidal plans were 2.3 times more prevalent in women with multimorbidity than in those without multimorbidity (OR, 95% CI: 2.30, 1.07–4.97 in model 2); however, this association was attenuated after further adjustment in model 3. Suicidal plans were 2.5 times more prevalent in middle aged people (41–64 years) with multimorbidity than in those without multimorbidity (OR, 95% CI: 2.54, 1.12–5.77 in model 3). On the other hand, there was no statistically significant association between multimorbidity and suicidal plans among younger or older men.

## 4. Discussion

In this study, it was shown that 30.8% of the total population of the 7th KNHANES has multimorbidity with more than two chronic diseases. As other studies have shown, the prevalence of multimorbidity increases with age, [19] with 60.4% of people aged 65 and older having multimorbidity. The proportion of women found with multimorbidity is overwhelmingly higher than that of men, which is the same as in previous studies [20]. Further, this study found evidence that level of education and smoking and drinking habits are related to multimorbidity.

We verified differences in suicidal thoughts and plans according to whether participants had multimorbidity. A comparative analysis was performed on these two groups drawing on data from the 2017 waves of the 7th KNHANES. Participants who had multimorbidity had a significantly greater prevalence of suicidal thoughts compared to those without such group of diseases. It was also found that the higher the number of chronic diseases, the higher the percentage of suicidal thoughts and suicidal plans.

As life extension continues to increase in South Korea, it has become necessary to change the perception of diseases among adults. This new understanding is essential for properly managing patients with multimorbidity [3]. Multimorbidity is highly likely to occur from polypharmacy, adverse drug side effects, or competing medical recommendations [21,22,23]. Moreover, if a person has multimorbidity, their risk of suicide appears to increase. The concurrence of chronic illnesses can lead to a decline in functional state and increase in mortality; therefore, more emphasis must be placed on the comprehensive impact of multimorbidity on patients’ overall health and quality of life [24].

When a stratified analysis was conducted by age, younger people with multimorbidity were more at risk of suicide than those without multimorbidity. In this study, the associations of multimorbidity with suicidal thoughts in older adults aged over 65 were lower than in other age groups and did not affect suicidal plans. However, the suicide rate among older adults is increasing as the population of this group increases. According to South Korea’s statistics for 2014, the suicide rate is approximately 37.5 per 100,000 people aged in their 60s, 57.6 per 100,000 people aged in their 70s, and 78.6 per 100,000 people aged in their 80s, which are all much higher than the rates of 17.8 and 27.9 of people in their 20s and 30s, respectively. These rates indicate that older adults have a higher risk of suicide compared to other age groups [25]. It is also relevant to mention that suicide attempts among older adults are more likely to lead to death compared to other age groups [25,26]. However, unlike suicide in other age groups, suicide among older adults is generally not impulsive, and diverse realistic factors tend to contribute to its occurrence [26,27].

When a stratified analysis was conducted by sex, there was a significant difference of suicidal thoughts according to multimorbidity in men and women. In this study, the associations of multimorbidity with suicidal thoughts in men are higher than in women. This shows that there is a difference in suicide between women and men, corroborating other studies [28,29,30]. However, suicidal plans according to multimorbidity in men and women did not significantly differ.

Although this study provides reliable basic data on multimorbidity and suicidal thoughts and plans in a representative sample of Koreans, it does not go without limitations. First, because of their nature, cross-sectional studies cannot explain causal relationships between disease status and the various suicide-related relevant variables among adults. Furthermore, we evaluated only the presence of suicidal thoughts in the last year, without considering the frequency of these thoughts. Second, because participants’ disease status was recorded based on self-reports, it is possible that biases affected the data. Third, we could not perform a detailed survey of assessed suicidal thoughts and plans, as we relied on previously written questions. In addition, while we might have considered some of the factors that influence suicidal thoughts, we could not account for all confounding variables. Finally, because older adults only accounted for a small proportion of all participants in the KNHANES, there was a major difference in the number of participants between disease groups. Therefore, there is the chance of false negatives and underestimation for some age-related results.

Previous studies have analyzed suicidal thoughts in relation to chronic illnesses; however, this study is unique in verifying whether participants had suicidal thoughts and plans according to whether they had multimorbidity. The presence of suicidal thoughts is a key risk factor for death by suicide. Suicidal behavior often recur in people who have previously attempted suicide, and suicide attempts often occur in people who frequently think about suicide [31,32]. Therefore, to prevent suicide, people with suicidal thoughts must be categorized as a high-risk group for suicide attempts and be cared for accordingly. By extension, it is necessary to consider multimorbidity in suicide prevention measures, as these individuals appear at risk of suicide. Interventions by medical professionals should, therefore, consider these thoughts as reflective of a person’s mental health status [33]. Four weeks before suicide death, about 50% visited medical instructions such as outpatients, inpatients, and emergency room visits, and 83% of them visited within one year [34]. Therefore, it is necessary to screen suicidal risk for high-risk patients.

In conclusion, early detection and intervention regarding suicidal thoughts is essential in suicide prevention strategies for older adults with multimorbidity. Further research is needed to determine the suicide-related risk by chronic diseases and any clusters of chronic diseases.

## Figures and Tables

**Table 1:** General characteristics of participants. Data are presented as % (standard error (SE)).

		Without Multimorbidity (*n* = 4007)		Multimorbidity (*n* = 1787)		*p*-Value
		*n*	% (SE)	*n*	% (SE)	
Age (years)	19–40	1531	91.7 (0.9)	135	8.3 (0.9)	<0.001
	41–64	1890	73.4 (1.0)	746	26.6 (1.0)	
	≥65	586	39.6 (1.5)	906	60.4 (1.5)	
Sex	Male	1836	77.2 (1.0)	723	22.8 (1.0)	<0.001
	Female	2171	72.4 (1.1)	1064	27.6 (1.1)	
Personal income	≤1st quartile	536	52.9 (1.7)	597	47.1 (1.7)	<0.001
	≥2nd quartile	3471	78.8 (0.8)	1190	21.2 (0.8)	
Education (years)	≤9	843	49.7 (1.4)	973	50.3 (1.4)	<0.001
	≥10	3164	82.4 (0.8)	814	17.6 (0.8)	
Smoking status	Never smoker	2491	74.0 (1.1)	1151	26.0 (1.1)	<0.001
	Former smoker	737	70.5 (1.6)	391	29.5 (1.6)	
	Current smoker	779	81.1 (1.4)	245	18.9 (1.4)	
Alcohol consumption	Non-drinker	1605	67.4 (1.2)	1019	32.6 (1.2)	<0.001
	Alcohol drinker	2402	79.9 (0.9)	768	20.1 (0.9)	

**Table 2:** Suicidal thoughts and plans according to the number of chronic diseases. Data are presented as % (standard error (SE)). * Obtained by using chi-square test.

		Number of Chronic Diseases				
		0 (*n* = 2348)	1 (*n* = 1659)	2 (*n* = 943)	≥3 (*n* = 844)	*p*-Value *
Suicidal thoughts (−)	*n*	2279	1589	877	761	<0.001
	% (SE)	97.3 (0.4)	95.5 (0.7)	92.5 (1.3)	90.3 (1.3)	
Suicidal thoughts (+)	*n*	69	70	66	83	
	% (SE)	2.7 (0.4)	4.5 (0.7)	7.5 (1.3)	9.7 (1.3)	
Suicidal plans (−)	*n*	2332	1637	925	824	0.002
	% (SE)	99.4 (0.2)	98.4 (0.4)	98.0 (0.5)	97.8 (0.5)	
Suicidal plans (+)	*n*	16	22	18	20	
	% (SE)	0.6 (0.2)	1.6 (0.4)	2.0 (0.5)	2.2 (0.5)	

**Table 3:** Suicidal thoughts and plans among participants with and without multimorbidities. Values are presented as weighted % (standard error (SE)). * Obtained by using chi-square test.

	Without Multimorbidity (*n* = 4007)		Multimorbidity (*n* = 1787)		*p*-Value *
	*n*	% (SE)	*n*	% (SE)	
Suicidal thoughts (−)	3868	96.6 (0.4)	1638	91.5 (1.0)	<0.001
Suicidal thoughts (+)	139	3.4 (0.4)	149	8.5 (1.0)	
Suicidal plans (−)	3969	99.0 (0.2)	1749	97.9 (0.4)	0.003
Suicidal plans (+)	38	1.0 (0.2)	38	2.1 (0.4)	

**Table 4:** Associations of multimorbidity and suicidal thoughts and plans. OR, odds ratio; CI, confidence interval. Model 1 was not adjusted. Model 2 was adjusted for sex and age. Model 3 was adjusted for sex, age, education, personal income, smoking status, and alcohol consumption. Values were calculated by multivariable logistic regression analysis.

	Suicidal Thoughts		Suicidal Plans	
	OR (95% CI)	*p*	OR (95% CI)	*p*
Total				
Model 1	2.65 (1.95–3.60)	<0.001	2.14 (1.30–3.54)	0.003
Model 2	2.49 (1.82–3.40)	<0.001	2.26 (1.22–4.20)	0.010
Model 3	2.14 (1.54–2.97)	<0.001	2.01 (1.08–3.73)	0.028
Sex				
Men				
Model 1	3.30 (2.06–5.28)	<0.001	2.33 (1.02–5.30)	0.044
Model 2	3.40 (2.05–5.65)	<0.001	2.29 (0.85–6.20)	0.101
Model 3	3.09 (1.84–5.20)	<0.001	2.00 (0.74–5.45)	0.172
Women				
Model 1	2.18 (1.57–3.04)	<0.001	1.97 (1.06–3.67)	0.032
Model 2	1.92 (1.33–2.76)	0.001	2.30 (1.07–4.97)	0.034
Model 3	1.63 (1.12–2.36)	0.011	2.01 (0.94–4.33)	0.073
Age (years)				
19–40				
Model 1	3.15 (1.50–6.60)	0.003	2.30 (0.61–8.68)	0.220
Model 2	3.14 (1.50–6.58)	0.003	2.34 (0.62–8.77)	0.207
Model 3	3.15 (1.43–6.97)	0.005	2.04 (0.45–9.18)	0.350
41–64				
Model 1	2.58 (1.65–4.04)	<0.001	3.37 (1.48–7.68)	0.004
Model 2	2.55 (1.63–4.00)	<0.001	3.34 (1.48–7.55)	0.004
Model 3	1.83 (1.17–2.85)	0.008	2.54 (1.12–5.77)	0.026
≥65				
Model 1	1.87 (1.21–2.90)	0.005	1.16 (0.47–2.84)	0.750
Model 2	1.89 (1.21–2.96)	0.005	1.20 (0.48–2.97)	0.697
Model 3	1.89 (1.20–2.98)	0.006	1.20 (0.47–3.10)	0.700
